# An Oncogenic Role for Four-Jointed Box 1 (FJX1) in Nasopharyngeal Carcinoma

**DOI:** 10.1155/2019/3857853

**Published:** 2019-05-19

**Authors:** San Jiun Chai, Muhammad Mamduh Ahmad Zabidi, Siew Pey Gan, Pathmanathan Rajadurai, Paul Vey Hong Lim, Ching Ching Ng, Lee Fah Yap, Soo Hwang Teo, Kue Peng Lim, Vyomesh Patel, Sok Ching Cheong

**Affiliations:** ^1^Cancer Research Malaysia, No. 1, Jalan SS12/1A, Subang Jaya, Selangor, Malaysia; ^2^Institute of Biological Sciences, Faculty of Science, University of Malaya, Malaysia; ^3^Department of Pathology, Subang Jaya Medical Center, Subang Jaya, Selangor, Malaysia; ^4^Western Medical Division, Tung Shin Hospital, 102 Jalan Pudu, Kuala Lumpur, Malaysia

## Abstract

Nasopharyngeal carcinoma (NPC) is a highly metastatic cancer prevalent in Southern China and Southeast Asia. The current knowledge on the molecular pathogenesis of NPC is still inadequate to improve disease management. Using gene expression microarrays, we have identified the *four-jointed box 1* (*FJX1*) gene to be upregulated in primary NPC tissues relative to nonmalignant tissues. An orthologue of human *FJX1*, the *four-jointed (fj)* gene in Drosophila and *Fjx1* in mouse, has reported to be associated with cancer progression pathways. However, the exact function of FJX1 in human is not well characterized. The overexpression of FJX1 mRNA was validated in primary NPC tissue samples, and the level of FJX1 protein was significantly higher in a subset of NPC tissues (42%) compared to the normal epithelium, where no expression of FJX1 was observed (*p* = 0.01). FJX1 is also found to be overexpressed in microarray datasets and TCGA datasets of other cancers including head and neck cancer, colorectal, and ovarian cancer. Both siRNA knockdown and overexpression experiments in NPC cell lines showed that FJX1 promotes cell proliferation, anchorage-dependent growth, and cellular invasion. Cyclin D1 and E1 mRNA levels were increased following FJX1 expression indicating that FJX1 enhances proliferation by regulating key proteins governing the cell cycle. Our data suggest that the overexpression of FJX1 contributes to a more aggressive phenotype of NPC cells and further investigations into FJX1 as a potential therapeutic target for NPC are warranted. The evaluation of FJX1 as an immunotherapy target for NPC and other cancers is currently ongoing.

## 1. Introduction

Nasopharyngeal cancer (NPC) is a highly metastatic cancer that is particularly prevalent in Southeast Asia and Southern China with an incidence rate of 20-50 per 100,000 persons per year [1, 2]. According to GLOBOCAN 2018, a total of 129,097 new cases of NPC and 72,987 death cases worldwide is estimated in 2018 [3]. Radiotherapy is effective against early-stage NPC; however, over 70% of cases present with late-stage disease and only 10-40% of these patients survive for more than 5 years [4, 5]. Currently, the mainstay treatment for locoregional advanced cases of NPC is concurrent chemoradiotherapy. Unfortunately, undesirable complications such as xerostomia, cranial nerve neuropathy, and osteoradionecrosis occur frequently after the treatment because of the location of the tumour at the base of the skull that is closely surrounded by and in close proximity to many vital structures such as the brain, spinal cord, eyes, ear, and parotid glands that result in high morbidity and poor quality of life [6, 7].

NPC is consistently associated with Epstein-Barr virus (EBV) infection [8], and it is well recognized that EBV alters many functional properties that are involved in tumour progression [9]. However, the exact contribution of EBV to the pathogenesis of NPC is not fully understood. The molecular events that drive the progression of NPC are still elusive, and it is likely that a better understanding of its molecular pathogenesis will lead to the identification of novel biomarkers and therapeutic targets.

High-throughput analyses such as microarrays and genome sequencing have facilitated the discovery of many potential biomarkers for diagnostics, therapeutics, and prediction of treatment outcome. Using platforms such as microarrays, whole genome sequencing, targeted deep sequencing, and SNP arrays, several important pathways such as ErbB-PI3K, Akt-mTOR, Notch, and NF-*κ*B were found to be frequently altered among NPC patients [10–12], suggesting that these pathways could be targeted for treating NPC.

Using expression microarrays, we have previously identified genes that are differentially expressed in EBV-positive primary NPC tumours compared to cancer-free nasopharyngeal tissue samples [13]. From this study, several potential oncogenes or candidate biomarkers such as FJX1, WNT5A, CLDN1, FGFR3, FZD6, RALA, and CLCA2 were shortlisted based on previous reports on their involvement in cancer pathophysiology, neoplasia, and embryogenesis process. Among these genes, the human four-jointed box 1 (FJX1) was chosen to be further characterized by its function in NPC development. In this study, we show that FJX1 is overexpressed at both the mRNA and protein levels in a subset of primary NPC tumours, as well as in other cancers. Following the knockdown of the gene in FJX1-overexpressed cells and overexpression of FJX1 in low-expressing cell lines, respectively, we demonstrated that this gene promotes the proliferation, anchorage-independent growth, and invasion of NPC cells. Together, our data suggest a possible role for FJX1 in the pathogenesis of NPC.

## 2. Materials and Methods

### 2.1. Cell Lines and Tissue Biopsies

The cell lines used in this study included HK1 and HeLa/T, a HeLa hybrid that was previously reported as a cell line derived from NPC (TW04). Cell line authentication showed that the STR profile of this line shared 80.0% similarity with D98-AH2, a clone of HeLa (Supplementary Table 1). The cell lines were cultured in RPMI-1640 (Gibco, USA), supplemented with 2 mM L-glutamine (Sigma-Aldrich, USA) and 10% fetal bovine serum (FBS) (Gibco, USA). Fresh-frozen nasopharyngeal biopsies (*n* = 16) from newly diagnosed and treatment-naïve patients from Tung Shin Hospital (Kuala Lumpur, Malaysia) were included in the quantitative real-time PCR analysis (qRT-PCR). Clinical information on these biopsies indicated that 14 were undifferentiated Epstein–Barr virus-encoded small RNA- (EBER-) positive NPC, while 2 were EBER-negative nasopharyngeal biopsies with no evidence of malignancy. A further 43 paraffin-embedded archival tissue samples from NPC patients diagnosed at Tung Shin Hospital, Kuala Lumpur, were obtained and included in our analysis. As controls, 11 archived tissue samples of the nonmalignant nasopharynx tissues with no evidence of NPC were obtained from the Tung Shin Hospital, Kuala Lumpur, and Nilai Cancer Centre, Negeri Sembilan (Supplementary Table 2). Ethical approval for this study was obtained from the independent ethics committee of respective institutions, and written informed consent was obtained from all patients before tissue collection.

### 2.2. Quantitative Real-Time PCR (qRT-PCR)

Transcriptomic levels of target antigens after knockdown assays were determined by qRT-PCR. Briefly, total RNA from fresh frozen biopsies was extracted using NucleoSpin® RNA II (Macherey-Nagel, Germany), and 1 *μ*g total RNA was subjected to reverse transcription using SuperScript II reverse transcriptase (Invitrogen, USA). The resulting cDNA was used as input to perform qRT-PCR to detect FJX1 and CCND1 expression at the transcription level using corresponding TaqMan probes (FJX1: Hs00534909_s1; CCND1: Hs00765553_m1; Applied Biosystems, US) and primer pairs (FJX1 5′-CCCGCAAAGGTGTCTAAAAACT-3′ and 5′GTGCTGGCACAGTAAAGAATCCT 3′; CCND1 5′-CCCTGACGGCCGAGAAG-3′ and 5′-GGTCTGCGCGTGTTTGC 3′). The transcriptional level of CCNE1 was assessed using SYBR Green (Life Technologies, US) with primer 5′-CTGGATGTTGACTGCCTTGAATT-3′ and 5′-GCGACGCCCCTGAAGTG-3′. The ABI Prism 7000 Sequence Detection System (Applied Biosystems, USA) was used for both the TaqMan and SYBR Green assays. In parallel, GAPDH (5′-GAAGGTGAAGGTCGGAGTC-3′ and 5′-GAAGATGGTGATGGGATTTC-3′) was included to serve as an internal control for normalization, and all experiments were performed in triplicate following the manufacturer's protocol. A no-template control was included to assess the overall specificity of the reaction. The nonmalignant nasopharynx tissue with the highest level of FJX1 was used to compare with FJX1 expression of NPC tissues. Fold changes in gene expression between nonmalignant nasopharynx tissue and tumour samples were measured using the comparative threshold cycle method (∆∆Ct), as described previously [13]. Compared to the controls, expression of the target genes was considered to be significantly upregulated when the fold change of expression > 2 with the *p* < 0.05 by a two-tailed *t*-test.

### 2.3. Immunohistochemistry

Immunohistochemistry (IHC) was performed to determine the expression levels and localization of FJX1 protein using Dako REAL™ EnVision Detection System (DakoCytomation, Denmark). Antigen retrieval was performed by microwaving the sections in high pH antigen retrieval solution (Link Technology, UK) for 30 minutes, and endogenous peroxidase activity was quenched using Dako REAL™ Peroxidase-Blocking Solution for 15 minutes. Tissue sections were washed with phosphate-buffered saline (PBS) and then incubated with anti-human FJX1 rabbit polyclonal antibody at 1 : 500 (Aviva Systems Biology, USA). After 1 hour of incubation at room temperature, the sections were washed once in Tris-buffered saline (TBS) buffer containing 0.1% Tween-20 (TBST) for 5 minutes, and this was further incubated with the HRP-conjugated polymer for 30 minutes. After washing for 5 minutes in TBST, the expression of FJX1 was visualized with the development of diaminobenzidine (DAB) as a chromogen, and the sections were counterstained with Mayer's haematoxylin (BDH Laboratories, UK), washed in tap water, dehydrated in graded alcohols, cleared in xylene, and mounted in DPX (Fluka BioChemika, USA). Immunoreactivity of epithelial cells (cancer and normal) was scored based on a 4-point intensity scoring system: 0 = negative expression, 1 = weak positive, 2 = moderate positive, and 3 = strong positive. The ductal epithelium of the NPC tissues which were always stained positive served as the internal positive control for IHC.

### 2.4. Knockdown and Overexpression of FJX1

To investigate if FJX1 drives the progression of NPC, HK1 which expresses a high level of FJX1 endogenously was selected in the knockdown assay, while HeLa/T cells with low levels of FJX1 were chosen in the overexpression study. To knockdown the expression of FJX1 expression in NPC cell line HK1, ON-TARGETplus SMARTpool siRNA targeting FJX1 (HK1-siFJX1) or Non-Targeting Pool siRNA (HK1-siNT) was used at 50 nM (Dharmacon, USA). Approximately, 7.5 × 10^4^ cells per well were seeded into 6-well plates, cultured overnight, and transfected with the relevant siRNA using DharmaFECT 1 transfection reagent (Dharmacon, USA). The FJX1 siRNA pool contained 4 sequences targeting different coding regions of FJX1, and the sequences include 5′-CGGAGCAGAUUCAGGGCGA-3′, 5′-AGUACAAUGGACCGACUUA-3′, 5′-UCGACUACCUGACGGCCA-3′, and 5′-GGACUUAGUGUCACCGGGA-3′. The effect of FJX1 knockdown was confirmed by qRT-PCR analysis as a good anti-FJX1 antibody is not available.

To overexpress FJX1 in the HeLa/T cells, firstly, the coding region of FJX1 was amplified from cDNA of NP460 cells by PCR using the GC-RICH PCR System (Roche, Germany) and PCR products after purification were cloned into the pcDNA3.1-V5/His-B vector using BamH1 and EcoR1 restriction sites. Approximately, 3 × 10^4^ HeLa/T cells per well were seeded into 6-well plates, cultured overnight, and then transfected with the plasmids using Lipofectamine™ 2000 transfection reagent (Invitrogen, USA). Cell lines overexpressing FJX1 are referred to as HeLa/T-FJX1, while cell lines transfected with vector control were referred to as HeLa/T-vector.

### 2.5. Western Blotting

To confirm the expression of FJX1 after transfection, transfected HeLa/T cells were lysed on ice in RIPA buffer (50 mM Tris pH8, 1% NP-40, 0.5% sodium deoxycholate, and 0.1% SDS, 150 mM NaCl) containing the protease inhibitor cocktail (Roche, USA). Cell lysates were collected after centrifugation at 14000 rpm for 15 minutes at 4°C, and the concentration of the total cellular protein for each sample was determined using the Bradford protein assay (Pierce Biotechnology, USA). Extracted proteins (50 *μ*g) were denatured at 95°C for 5 minutes followed by a 12% SDS-polyacrylamide gel separation, and separated proteins were transferred onto the Immobilon-P membrane (Millipore, MA, USA). Then, blots were blocked with 5% skimmed milk in TBST for 1 hour and further incubated with the indicated primary antibodies (anti-V5: 1 : 2000; Abcam, UK; anti-*α*-tubulin: 1: 2000; Sigma-Aldrich, USA) overnight at 4°C. The next day, blots were washed (3 times for 5 minutes each) in TBST buffer and subsequently incubated with the relevant IRDye Odyssey secondary antibodies (LI-COR Biosciences, USA) for 1 hour. After washing (TBST), detection was performed using the Odyssey Infrared Imaging System (LI-COR Biosciences, USA). Due to the unavailability of a good antibody against FJX1, an anti-V5 antibody was used to detect FJX1 overexpression after transfection.

### 2.6. Cell Proliferation Assay

To determine the role of FJX1 in promoting cell proliferation, HeLa/T-FJX1 and HeLa/T-vector or HK1-siFJX1 and HK1-siNT were seeded at 5 × 10^4^ cells per well in 6-well plates in triplicate, 24 hours post-transfection. The cells were trypsinized everyday for 5 days and counted on a CASY cell counter (Roche Innovatis AG, Germany). The mean cell counts and standard errors were calculated.

### 2.7. Soft-Agar Colony Formation Assay

To determine the ability of FJX1 overexpression to drive cellular anchorage-independent growth *in vitro*, low-melting agarose VII (Sigma-Aldrich, USA) was used in the colony formation assays. Approximately, 24 hours post-transfection with plasmids, single-cell suspensions of 2 × 10^3^ cells per well were plated into 6-well plates in 2 mL of RPMI containing 10% FBS and 0.5% agar on a layer of 2 mL of the same medium containing 1% agar. After 3 weeks, colonies were counted microscopically. The mean colony counted from each field and standard errors were calculated.

### 2.8. *In Vitro* Invasion Assay

Invasion assays were carried out using Matrigel-coated 8 *μ*m pore size polycarbonate Transwell filters (Corning, USA) as described previously [14]. Approximately 24 hours post-transfection with siRNAs targeting FJX1 or FJX1 expression plasmids, cells were treated with 15 *μ*g/mL mitomycin C (Sigma-Aldrich, USA) for two hours to inhibit proliferation. Approximately 5 × 10^4^ cells in 200 *μ*L migration buffer (0.1% BSA/serum-free RPMI) were plated into the upper chamber and left to incubate for 48 hours. Total cells having invaded to the lower chamber were trypsinized and counted using the CASY cell counter (Roche Innovatis AG, Germany).

### 2.9. Bioinformatic Analysis

Microarray data from the Gene Expression Omnibus (GEO) repository [15] and the datasets that compared gene expression levels in NPC and colorectal and ovarian tumours to normal tissues from the respective sites were analysed. For the RNA-Seq data, transcriptomic data from 43 head and neck cancer tissue samples and matched normal samples from TCGA database [16] were analysed.

### 2.10. Statistical Analysis

For the immunohistochemical analysis, the comparison between groups was determined by Fisher's exact test. For *in vitro* studies, statistical differences between experimental groups were evaluated by two-tailed *t*-test and *p* < 0.05 was taken as being statistically significant.

## 3. Results

### 3.1. FJX1 Is Overexpressed in NPC

**Figure 1:**
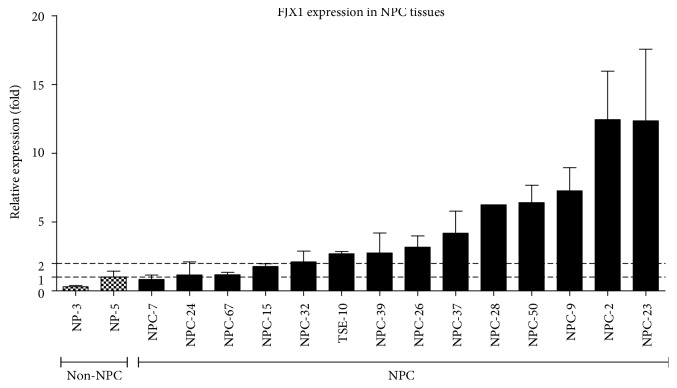
Overexpression of FJX1 in NPC. Quantitative PCR showed that when compared with two biopsies of the normal nasopharynx (NP-3 & NP-5), FJX1 significantly elevated at transcriptomic levels in 10 of the 14 samples that showed significant upregulation by array analysis.

We first sought to confirm that FJX1 is overexpressed in NPC, having previously shown that FJX1 was significantly upregulated in 14/25 (56%) cases by microarray analysis (Supplementary Figure S1a) [13]. Fourteen samples that were from the previous microarray analysis were available for qRT-PCR analysis, and 10/14 demonstrated at least a 2-fold increase in FJX1 levels when compared with the two nonmalignant controls (Figure 1).

**Figure 2:**
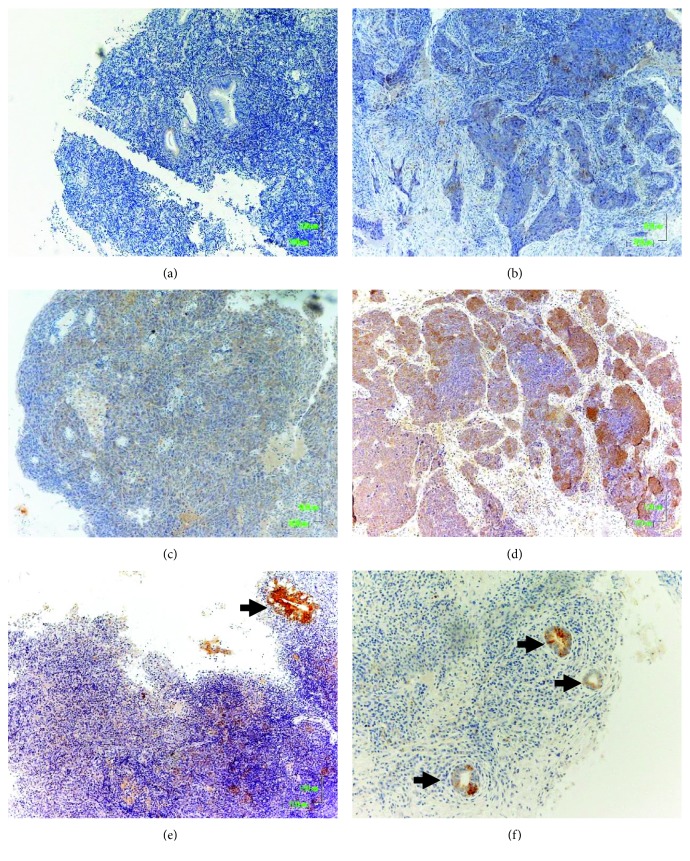
Immunohistochemistry confirms the upregulation of FJX1 protein expression in NPC, but not in nonmalignant nasopharynx tissues. Representative images of (a) nonmalignant tissues and (b)–(d) NPC samples with a staining intensity of 1-3 are shown. (e) and (f) Endothelial ductal served as an internal positive control for the staining.

The expression and cellular localization of FJX1 protein were further examined in an independent set of 43 formalin-fixed paraffin-embedded primary NPC and 11 nonmalignant nasopharyngeal samples by immunohistochemistry (Figure 2). The normal epithelium in the control samples and the normal epithelium adjacent to carcinoma were consistently negative for the expression of FJX1 in all 11 nonmalignant samples (Figure 2(a)). FJX1 expression was detected in 18 of 43 (42%) of the tumours examined, consistent with our microarray and qRT-PCR observations that FJX1 is upregulated in NPC. The expression of FJX1 was cytoplasmic, and the intensity ranged from weak to strong (Figures 2(b)–2(d)). The ductal epithelium of the nasopharynx tissues served as an internal positive control and consistently stained positive for FJX1 (Figures 2(e) and 2(f)). There was no significant association between the expression of the FJX1 protein and the clinical parameters (T, N, and overall stage) (Supplementary Table 3).

As a dysregulated gene in one cancer is typically also dysregulated in other types of cancer [17], we sought to explore whether this is also the case for FJX1. Indeed, besides our own microarray dataset (GSE13597), we found that FJX1 is also overexpressed in independent NPC microarray dataset (GSE12452) (2.8 fold), as well as colorectal and ovarian cancers [18, 19] (2.4- and 2.3-fold, respectively; *p* < 0.01) when compared to normal tissues (Table 1). Notably, RNA-Seq data from 43 head and neck cancers with matched normals from the TCGA database [16] consistently showed that tumour samples had a 2-fold elevated level of FJX1 when compared to the matched normals (*p* < 0.01) (Supplementary Figure S1b).

### 3.2. FJX1 Promotes NPC Cell Proliferation through Cell Cycle Regulation

**Figure 3:**
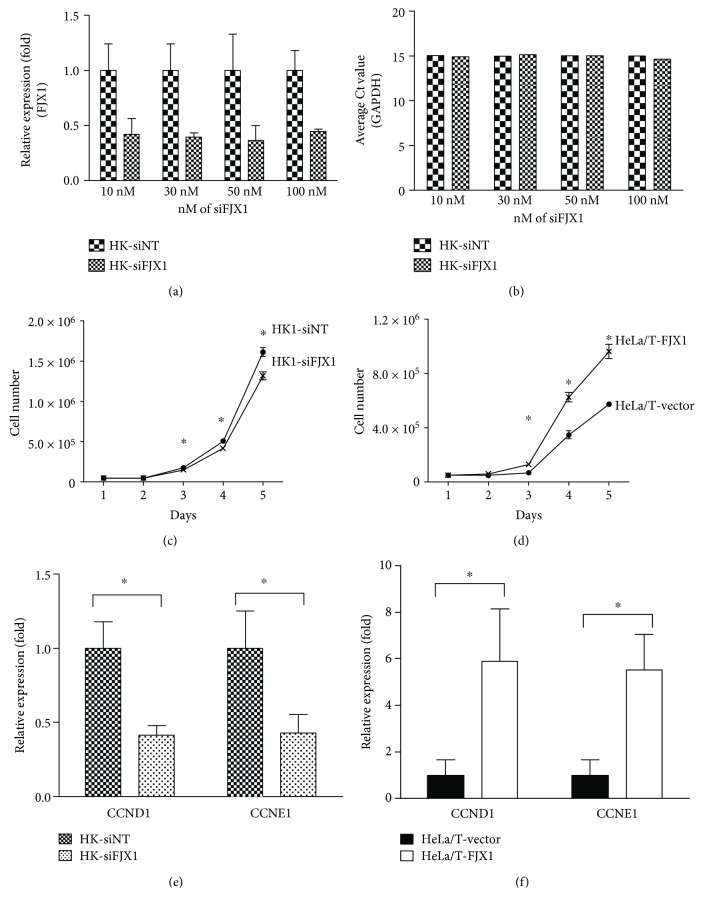
FJX1 promotes cell proliferation. (a) HK1 cells transfected with different concentrations of nontargeting (siNT) or FJX1-specific (siFJX1) siRNA were analysed by qRT-PCR. (b) Transfected HK1 cells with various concentrations of siFJX1 do not affect the expression of the housekeeping gene GAPDH. (c) HK1 cells transfected with FJX1 siRNA grew significantly slower than control cells, (d) while overexpression of FJX1 in HeLa/T cells resulted in significantly increased cell proliferation. The mRNA levels of cyclin D1 and cyclin E1 were determined in (e) HK1 and (f) HeLa/T cells by qRT-PCR. Reduced levels of cyclin D1 and E1 were evident following the knockdown of FJX1 in HK1 cells, whereas HeLa/T cells expressing FJX1 showed higher levels of these two cyclins compared to the vector control cells. The expression levels of the controls were normalized to 1 (∗ denotes *p* < 0.05).

To investigate the phenotypic impact of FJX1 *in vitro*, HK1 cells which express high levels of FJX1 were transfected with FJX1 siRNAs. Two days following siRNA transfection, FJX1 levels were reduced by ~60% while the expression of GAPDH remained unaffected, indicating the specificity of the FJX1 siRNA (Figures 3(a) and 3(b)). On the other hand, a HeLa hybrid NPC cell line, HeLa/T, which expresses low levels of FJX1 was transfected with pcDNA3.1-V5/FJX1 and the expression confirmed by Western blotting. Three FJX1-specific bands of different molecular weights at around 50 kDa were clearly detected in the Western blotting analysis using a V5 antibody, potentially reflecting differential posttranslational modifications (Supplementary Figure S2). Previous study showed that two conserved potential N-linked glycosylation sites in the mouse are also present in human FJX1 [20]. A similar observation and multiple glycosylation sites in human FJX1 protein were also observed by Al-Greene et al. [18]. The occurrence of multiple bands on Western blotting could be due to the existence of an unglycosylated form as well as two other different forms of glycosylation at single or multiple sites.

Next, proliferation assays were performed on these two lines over 5 days. For HK1 cells with reduced FJX1 protein levels, the growth was consistently slower when compared to the HK1 cells transfected with siNT (*p* = 0.03). In contrast, HeLa/T with elevated levels of FJX1 grew significantly faster (*p* = 0.01), when compared with their respective control cells (Figures 3(c) and 3(d)). The data suggest that high FJX1 levels promote cell proliferation. To examine whether FJX1 enhances cell proliferation through cell cycle regulation, the expression of two molecules crucial to cell cycle progression, cyclin D1, and cyclin E1, were investigated. mRNA levels of cyclin D1 and E1 were reduced by approximately half following the knockdown of FJX1 in HK1 cells but were 5-fold elevated in FJX1-overexpressing HeLa/T cells (Figures 3(e) and 3(f)). These data suggest that FJX1 could promote cell growth by increasing the expression of positive regulators of cell cycle progression.

### 3.3. FJX1 Enhances Anchorage-Independent Growth

**Figure 4:**
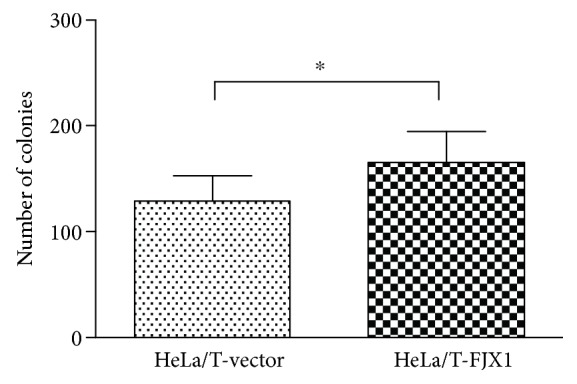
FJX1 enhances anchorage-independent growth. Compared to the control cells, the overexpression of FJX1 in HeLa/T cell lines resulted in significantly increased numbers of colonies (∗ denotes *p* < 0.05).

Soft agar colony formation assays were performed to determine the contribution of FJX1 to anchorage-independent cell growth, one of the most reliable markers of malignant transformation [21]. Comparing HeLa/T cells overexpressing FJX1 to those transfected with vector alone, we demonstrated that a high expression of FJX1 resulted in a significant increase in the number of colonies formed (*p* = 0.04; Figure 4), suggesting that FJX1 can contribute to a more aggressive phenotype in cancer. This assay was not performed on HK1 cells as these cells inherently do not form colonies in soft agar.

### 3.4. FJX1 Promotes NPC Cell Invasion

**Figure 5:**
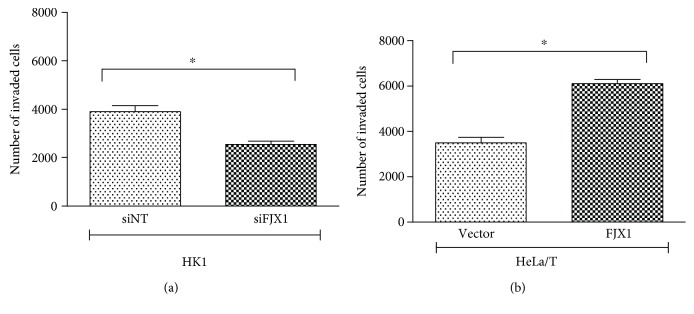
FJX1 promotes cell invasion. Inhibition of FJX1 in HK1 cells resulted in a 35% reduction in the number of cells invading compared to controls. By contrast, FJX1-expressing HeLa/T cells were significantly more invasive than the vector control cells (∗ denotes *p* < 0.05).

To examine the role of FJX1 in cell invasion, *in vitro* Matrigel Transwell assays were performed. As shown in Figure 5, 24 hours post-transfection with siFJX1 and siNT, the invasive ability of HK1 cells was reduced by ~35% following FJX1 knockdown (*p* = 0.02). Similarly, HeLa/T-FJX1 cells were also significantly more invasive than the vector control cells (*p* = 0.01). The capability of FJX1-expressing cells to invade through the Transwell membrane suggests that FJX1 enhances the invasiveness of tumour cells and could contribute to the ability of tumour cells to metastasize.

## 4. Discussion

In this study, we identified FJX1 to be overexpressed in NPC, and exogenous expression of FJX1 conferred a survival advantage to cancer cells. While much remains unknown about the function of FJX1, emerging studies have identified FJX1 to be amplified or overexpressed in several types of cancers including breast [22, 23], lung [24], ovarian [19], colorectal [18], and head and neck cancers [25, 26], suggesting that it could be a genetic driver for cancer. FJX1 is also found to be overexpressed in endometrium tissues from women with endometriosis [27]. Although endometriosis is considered a nonmalignant condition, the characteristics of endometriosis such as infiltration of endometriosis tissues to surrounding organs and presence of angiogenesis resembles a tumour-like behavior to some extent [18, 28, 29]. Microarray studies including ours demonstrated that *FJX1* is overexpressed in NPC relative to noncancerous tissues [13, 30, 31]. Overexpression of *FJX1* in the transcriptomic level was also observed in RNA-Seq data from 43 head and neck cancers compared to matched normal samples from the TCGA database. Further, *FJX1* amplification was also detected in 16/16 oral cancer cell lines derived from oral squamous cell carcinoma when compared to normal keratinocytes [32]. Taken together, these independent datasets demonstrate that FJX1 is consistently upregulated in cancers, particularly in head and neck cancer, underscoring the rationale of studying the function of this gene in these cancers.

Embryogenic development and cancer progression shared many similar characteristics. For example, during the developmental process, cells within the embryo go through rapid proliferation, placental implantation (invasion), cellular movement (migration), and formation of blood vessels (angiogenesis) [33, 34]. Further, genes and pathways which are tightly regulated in embryonic development are often found to be dysregulated or reactivated in cancers [35, 36]. Studies have shown the involvement and importance of four-jointed (Fj) protein in the developmental processes of various organisms. In *Drosophila*, Fj (the Drosophila equivalent of FJX1) is required for proximodistal growth and regulation of planar polarity where Fj mutant flies have shorter wings and legs compared with those that are wild-type [37, 38], while in crickets, Fj is involved in the regeneration of limbs by restoring the growth of amputated legs to normal size [39]. Similar observations were also reported in chick embryo development, where the chick *four-jointed* (*fjx*) expression was detected in limb buds at different phases and functions by first controlling the limb outgrowth then tissue differentiation at a later stage [40]. In mouse, Fjx1 is found in the developing brain and homozygous Fjx1 mutant mice showed abnormal morphology of dendrite arbors in the hippocampus of the brain. When cultured *in vitro*, hippocampal neurons from these mutant mice showed a higher level of dendrite extension and branching [41]. These studies underscore the importance of FJX1 in the development of different organisms. Although the exact function of FJX1 in human is not fully understood, data from the developmental studies suggests that the function of FJX1 in cancer development could be linked to its role in promoting growth and cell movement.

The human FJX1 protein shares 29% and 88% identity with its orthologue in *Drosophila* and mouse, respectively [42]. Fjx1 is a transmembrane kinase which is also known as the Golgi kinase four-jointed, Fj. Several studies on the orthologue of FJX1, the Fj, or Fjx1 in *Drosophila* and mouse Fjx1 revealed that Fjx1 is Notch-induced and plays a role in several important pathways including the Fat/Hippo pathway. Signaling pathways such as the Hippo, Wnt, Hedgehog, and Notch pathways were reported to be dysregulated and confer to the progression of many cancers [41, 43]. These pathways have already been shown to be important for head and neck cancers [16, 44]. Our study has revealed that the expression of FJX1 has a significant correlation of cancer cell proliferation and enhances anchorage-independent growth. A recent study showed that FJX1 is one of the downstream effectors of the frequently altered Hippo signaling pathway and FJX1 is a direct target of the YAP and TAZ oncogenes within this pathway [45].

Functionally, the Hippo pathway plays a critical role in regulating cell proliferation and apoptosis [46–48]. For the first time, we demonstrate that FJX1 plays a role in controlling cell proliferation and this is associated with an upregulation of cyclins including cyclin D1 and E1. Of note, Fjx1 has also been shown to form a feedback loop where overexpression of the protein further regulates the activation of YAP [49, 50].

In this study, we also demonstrated that FJX1 could increase the invasive potential of cancer cells. This phenotype has been shown *in vivo* in a non-small cell lung cancer model where Fjx1 overexpression was found to be associated with increased metastasis [51]. FJX1 could potentiate invasion perhaps through its involvement in regulating planar cell polarity (PCP), a process where cells orientate themselves within the tissue plane for collective cell movement [52]. While PCP is a critical process in wound repair and development, facilitating processes such as gastrulation, neural tube closure, and heart morphogenesis, to name a few, dysregulation could lead to pathological conditions including cancer invasion and metastasis. PCP is coordinated by Fat and Dachsous where these proteins are proposed to physically interact in a heterotypic fashion to mediate PCP [53]. Fj regulates the phosphorylation state of the cadherin repeats of Fat and Ds affecting their ability to interact with one another [37, 53–55] which could explain why upregulation of FJX1 results in increase invasion in head and neck cancer cells.

## 5. Conclusions

In summary, we demonstrated that FJX1 confers a survival advantage and invasive potential to nasopharyngeal carcinoma and is a likely target for the treatment of cancer. Its involvement in multiple important pathways controlling tumour growth and cell movement rationalises the development of targeted therapies against this protein. An *in vitro* study on the development of immunotherapy using peptides derived from FJX1 as a treatment for head and neck cancer including NPC has shown the ability of these peptides to elicit patients' T cell cytotoxic response towards tumour cells expressing FJX1 [56, 57]. The efficacy and safety of FJX1 peptide vaccine are currently under investigation using an *in vivo* mouse model.

## Figures and Tables

**Table 1:** Microarray datasets that reported the overexpression of FJX1.

GEO reference	Type of cancer	Tumour sample	Normal sample	Fold change	*p* value
GSE13597	NPC	25	3	1.89	0.04
GSE12452	NPC	31	10	2.82	8.73*E* − 07
GSE32323	Colorectal	17	17	2.44	2.49*E* − 06
GSE14407	Ovarian	12	12	2.23	0.000629

## Data Availability

The data used to support the findings of this study are available from the corresponding author upon request.

## Supplementary Materials

Supplementary Materials Table S1: STR profile of TW04 with a match of ≥80.0% to HeLa D98-AH2 clone. Table S2: histological characteristics of nonmalignant nasopharyngeal tissue samples. Table S3: correlation between FJX1 expression and clinicopathological characteristics of NPC tissue samples. Figure S1: transcriptomic levels of FJX1 in cancer. (a) Previous microarray analysis (GSE13597) showed that the mRNA level of the FJX1 transcript is increased in NPC biopsies and NPC cell lines compared to normal nasopharynx tissue, suggesting FJX1 as a potential biomarker for NPC. (b) FJX1 mRNA levels also reported being elevated in head and neck cancer patients when comparing RNA-Seq data of 43 cancer tissues with matched normal samples from the TCGA database. Figure S2: FJX1:V5 overexpression in HeLa/T cell was confirmed using the V5 antibody. Three bands with different molecular weights of FJX1 protein were detected in Western blotting.
