# Supplementary material for: An Oncogenic Role for Four-Jointed Box 1 (FJX1) in Nasopharyngeal Carcinoma
Source: Dis Markers. 2019 May 19;2019:3857853. doi: 10.1155/2019/3857853 (PMC6545767; doi:10.1155/2019/3857853)

An oncogenic role for Four-jointed Box 1 (FJX1) in nasopharyngeal carcinoma

San Jiun Chai[1]*, Muhammad Mamduh Ahmad Zabidi[1,4], Siew Pey Gan[1], Pathmanathan Rajadurai[2], Paul Vey Hong Lim[3], Ching Ching Ng[4], Lee Fah Yap[1], Soo Hwang Teo[1], Kue Peng Lim[1], Vyomesh Patel[1], Sok Ching Cheong[1]*

Supplementary Tables

Supplementary Table 1. STR profile of TW04 with a match of ≥ 80.0% to HeLa D98-AH2 clone.

Type	Test Sample	Closest Match
**Cell Line**	**TW04**	**D98-AH2**
**Database**	**-**	**DSMZ**
TH01	6,7,9	7
D21S11	27,30	N/A
D5S818	11,12	11,12
D13S317	10,12	N/A
D7S820	10,12	12
D16S539	9,10	9,10
CSF1PO	10,11	9,10,11
Amelogenin	X	X
vWA	14,16	16,18
TPOX	8,12	8,12
**Match to Test Sample**		**80.00%**

Supplementary Table 2. Histological characteristics of non-malignant nasopharyngeal tissue samples.

Sample	Histology
NP 3	Nasopolyps
NP 4	Nasopharyngitis
NP 5	Normal epithelium
NP 6	Synovial sarcoma
NP 7	Nasopharyngitis
NP 8	Nasopharyngitis
NP 9	Nasopharyngitis
NP 10	Nasopharyngitis
NP 11	Adenoid
NP 12	Nasopharyngitis
NP 13	Lymphoid hyperplasia and rhinosinusitis

Supplementary Table 3. Correlation between FJX1 expression and clinicopathological characteristics of NPC tissue samples.

**Characteristics**	**FJX1 expression**	***p*-value**
T category		
T1	3/6 (50%)	0.948
T2	8/19 (42%)	
T3	4/12 (33%)	
T4	3/6 (50%)	
N category		
N0	5/12 (42%)	0.841
N1	4/6 (67%)	
N2	9/23 (39%)	
N3	0/2 (0%)	
Stage		
I	2/3 (67%)	0.526
II	4/7 (57%)	
III	9/24 (38%)	
IV	3/9 (33%)	

Supplementary Figure Legends

Figure S1. Transcriptomic levels of FJX1 in cancer. (a) Previous microarray analysis (GSE13597) showed that the mRNA level of FJX1 transcript is increased in NPC biopsies and NPC cell lines compared to normal nasopharynx tissue, suggesting FJX1 as a potential biomarker for NPC. (b) FJX1 mRNA levels also reported being elevated in head and neck cancer patients when comparing RNAseq data of 43 cancer tissues with matched normal samples from the TCGA database.

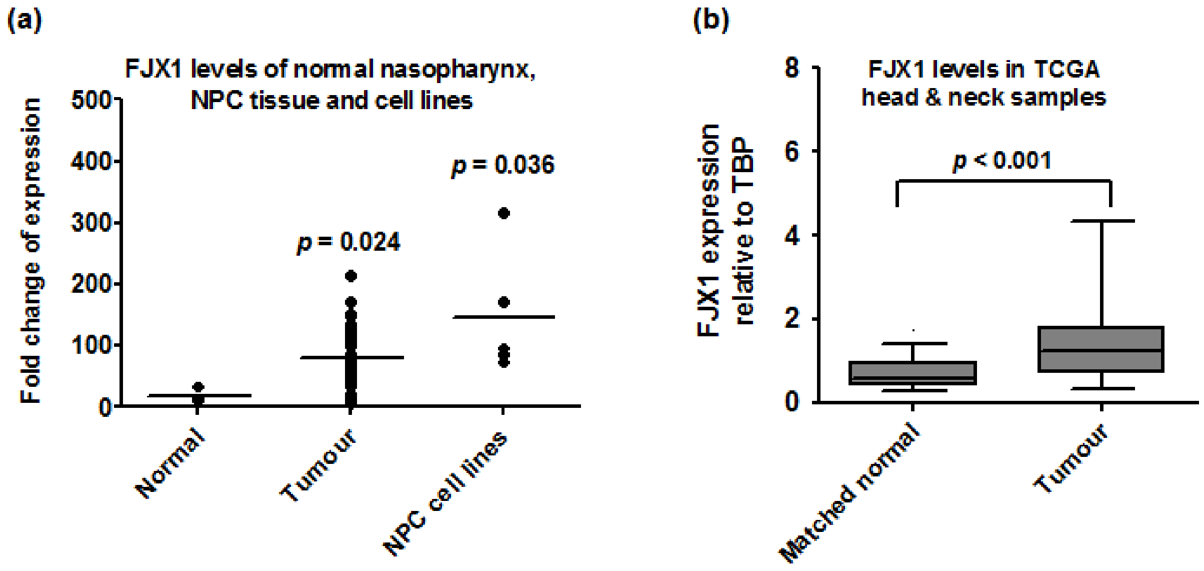

Figure S2. FJX1:V5 overexpression in HeLa/T cell was confirmed using V5 antibody. Three bands with different molecular weight of FJX1 protein in was detected in Western blotting.

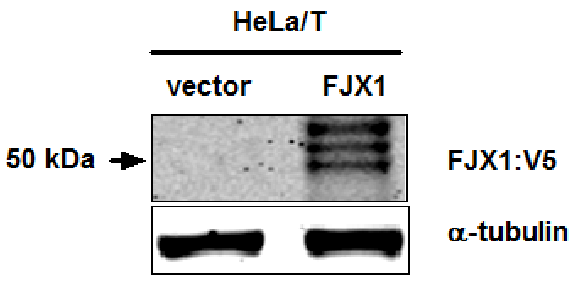

Supplement: Supplementary Materials — Table S1: STR profile of TW04 with a match of ≥80.0% to HeLa D98-AH2 clone. Table S2: histological characteristics of nonmalignant nasopharyngeal tissue samples. Table S3: correlation between FJX1 expression and clinicopathological characteristics of NPC tissue samples. Figure S1: transcriptomic levels of FJX1 in cancer. (a) Previous microarray analysis (GSE13597) showed that the mRNA level of the FJX1 transcript is increased in NPC biopsies and NPC cell lines compared to normal nasopharynx tissue, suggesting FJX1 as a potential biomarker for NPC. (b) FJX1 mRNA levels also reported being elevated in head and neck cancer patients when comparing RNA-Seq data of 43 cancer tissues with matched normal samples from the TCGA database. Figure S2: FJX1:V5 overexpression in HeLa/T cell was confirmed using the V5 antibody. Three bands with different molecular weights of FJX1 protein were detected in Western blotting. [file 3857853.f1.pdf]
